# Failure to release from the ER translocon induces 60S ribosome degradation

**DOI:** 10.64898/2026.09.26.754687

**Published:** 2026-09-28

**Authors:** Zebulon G. Levine, Stuti Khandwala, Christina J. Zhang, Kelsey L. Hickey, Julia Joung, Jingjie Hu, Yi Hua Chen-Neumann, Jingchuan Luo, Anders S. Hansen, J. Wade Harper, Gavin Schlissel, Jonathan S. Weissman

## Abstract

Ribosome malfunction has toxic consequences, but how mammalian cells select ribosomal subunits for degradation remains poorly understood. Here, we develop a pulse-chase CRISPR screening strategy to identify regulators of ribosome turnover and uncover that loss of protein UFMylation promotes ribosome degradation. Normal UFMylation releases the 60S subunit from the endoplasmic reticulum (ER) translocon. Without UFMylation, single-molecule tracking and optogenetic proximity labeling reveal accumulation of ER-associated 60S subunits and their specific degradation. This translocon-associated large subunit degradation (TLSD) is independent of the lysosome and, in our human cell model, is only active in nondividing, contact-inhibited cells. Degradation requires the poorly characterized protein R3HDM4, which we term TLSD1. Upon UFMylation loss, TLSD1 redistributes to the ER and promotes 60S degradation. These findings identify a cell-state-dependent pathway for clearing ER-retained 60S subunits. We propose translocon release acts as a checkpoint for ribosome function, explaining TLSD1’s genetic links to neuronal health and erythropoiesis.

## Full Text Availability

The license terms selected by the author(s) for this preprint version do not permit archiving in PMC. The full text is available from the preprint server.

